# Non-medical Cannabis Self-Exposure as a Dimensional Predictor of Opioid Dependence Diagnosis: A Propensity Score Matched Analysis

**DOI:** 10.3389/fpsyt.2018.00283

**Published:** 2018-06-27

**Authors:** Eduardo R. Butelman, Angelo G. I. Maremmani, Silvia Bacciardi, Carina Y. Chen, Joel Correa da Rosa, Mary Jeanne Kreek

**Affiliations:** ^1^Laboratory on the Biology of Addictive Diseases, Rockefeller University, New York, NY, United States; ^2^“VP Dole” Dual Diagnosis Unit, Azienda Ospedaliera-Universitaria Pisana, Pisa, Italy; ^3^Center for Clinical and Translational Science, The Rockefeller University Hospital, Rockefeller University, New York, NY, United States

**Keywords:** opioid, cocaine, cannabis, heroin, alcohol, dimensional, exposure, adolescence

## Abstract

**Background:** The impact of increasing non-medical cannabis use on vulnerability to develop opioid use disorders has received considerable attention, with contrasting findings. A dimensional analysis of self-exposure to cannabis and other drugs, in individuals with and without opioid dependence (OD) diagnoses, may clarify this issue.

**Objective:** To examine the age of onset of maximal self-exposure to cannabis, alcohol, cocaine, and heroin, in volunteers diagnosed with OD, using a rapidly administered instrument (the KMSK scales). To then determine whether maximal self-exposure to cannabis, alcohol, and cocaine is a dimensional predictor of odds of OD diagnoses.

**Methods:** This outpatient observational study examined maximal self-exposure to these drugs, in volunteers diagnosed with DSM-IV OD or other drug diagnoses, and normal volunteers. In order to focus more directly on opioid dependence diagnosis as the outcome, volunteers who had cocaine dependence diagnoses were excluded. Male and female adults of diverse ethnicity were consecutively ascertained from the community, and from local drug treatment programs, in 2002–2013 (*n* = 574, of whom *n* = 94 had OD diagnoses). The age of onset of maximal self-exposure of these drugs was examined. After propensity score matching for age at ascertainment, gender, and ethnicity, a multiple logistic regression examined how increasing self-exposure to non-medical cannabis, alcohol and cocaine affected odds of OD diagnoses.

**Results:** Volunteers with OD diagnoses had the onset of heaviest use of cannabis in the approximate transition between adolescence and adulthood (mean age = 18.9 years), and onset of heaviest use of alcohol soon thereafter (mean age = 20.1 years). Onset of heaviest use of heroin and cocaine was detected later in the lifespan (mean ages = 24.7 and 25.3 years, respectively). After propensity score matching for demographic variables, we found that the maximal self-exposure to cannabis and cocaine, but not to alcohol, was greater in volunteers with OD diagnoses, than in those without this diagnosis. Also, a multiple logistic regression detected that increasing self-exposure to cannabis and cocaine, but not alcohol, was a positive predictor of OD diagnosis.

**Conclusions/Importance:** Increasing self-exposure to non-medical cannabis, as measured with a rapid dimensional instrument, was a predictor of greater odds of opioid dependence diagnosis, in propensity score-matched samples.

## Introduction

Addictions to heroin or illicitly used prescription opioids (short-acting MOP-r agonists) cause major morbidity and mortality (1, 2), and there is considerable poly-drug use in persons with these diseases (3–6). Use of other substances, especially cannabis and alcohol, often precedes non-medical use of MOP-r agonists. The impact of non-medical cannabis use with respect to vulnerability to develop an opioid use disorder remains under study (7). This has been examined primarily with categorical classifications of cannabis use, such as “any use” vs. “no use” or presence vs. absence of a diagnosed cannabis use disorder. For example, epidemiological studies have shown that any cannabis use is associated with a later increase in odds of non-medical use of opioids and other drugs (8, 9). Another recent report from the NESARC longitudinal study found that any use of cannabis at the “wave 1” time point (2001–2002), was a positive predictor of both non-medical prescription opioid use, and opioid use disorder at the “wave 2” time point (2004–2005) (10).

In this study, we focus on dimensional aspects of drug self-exposure and their relationship to an opioid dependence diagnosis (OD). Dimensional measures are those that characterize a behavioral or biological variable along some form of a continuum. Specifically, we examined the ages of onset of heaviest use of different drugs in volunteers with opioid dependence diagnosis, as well as the level of maximal self-exposure. Dimensional aspects of substance use disorders (SUDs) are receiving recent attention, both for examination of disease progression and for the examination of mechanistic and genetic features (11–13).

Intriguingly, some studies have found that state-wide availability of medical cannabis has resulted in decreases in age-adjusted opioid overdose mortality (14), and other apparent protective effects (15). Experimental studies in humans do not detect a protective effect of the main psychoactive component of cannabis (the CB-1 partial agonist Δ9-tetra-hydro-cannabinol; Δ9-THC) on MOP-r agonist-induced respiratory depression, which is the underlying cause of overdose mortality (16). Also, cannabis smoking produced a small but significant increase in the abuse potential of a MOP-r agonist, in a recent laboratory study (17). Studies on the effectiveness of Δ9-THC in decreasing severity of withdrawal from MOP-r agonists have yielded mixed results, possibly due to different methods used (18, 19).

Some preclinical data show that exposure to Δ9-THC in adolescence can increase vulnerability to the addiction-related effects of MOP-r agonists in adulthood (20–22). Some, but not all, preclinical studies suggest that greater exposure to a CB1-r agonist could cause neurobiological effects that increase subsequent vulnerability to addictive-like effects of MOP-r agonists (23–25).

At least two major theories have been proposed to account for the sequence of first use of drugs, and also for specific patterns of poly-drug exposure in persons with specific SUD. Two of these major theories have been termed the “gateway theory” and the “shared vulnerability theory,” and their relative impact remains an area of controversy (6, 26–28). An examination of dimensional, as opposed to categorical, aspects of drug self-exposure could also provide a framework to further understand the aforementioned phenomena (11, 29).

Given the changes in cannabis availability and use, and the ongoing epidemic of opioid use disorders, this controversy is of current importance (30, 31). It has been suggested that dimensional data at the individual level would be of value to address this issue (32–34). However, few studies have examined dimensionally, how exposure to non-medical cannabis and other drugs can affect odds of a clinically diagnosed opioid use disorder, at the individual level (10, 35–37). Furthermore, in most studies where such data was examined, the instruments used are not suitable for general clinical or preventive practice, due primarily to their length. In this study, we therefore examined dimensionally how different amounts of self-exposure to major drugs of abuse including non-medical cannabis and alcohol, affected odds of developing an opioid dependence diagnosis, using a relatively rapid and simple instrument (38, 39).

## Materials and methods

This was an observational study, with consecutively ascertained adult volunteers who were examined in an outpatient research hospital setting, in the New York City area. This cohort was originally recruited and ascertained as part of genetic association studies of SUD (40–42).

### Volunteers

The main outcome under examination was the presence or absence of a DSM-IV opioid dependence (OD) diagnosis. Many of the volunteers with SUD also had other diagnoses in addition to OD, but the presence of a cocaine dependence diagnosis was an exclusion criterion for this study. However, volunteers with the relatively less severe DSM-IV diagnosis of cocaine *abuse* were not excluded. Volunteers were ascertained sequentially from a number of addictive disease treatment clinics in the greater New York City area, and from the local community in the same area.

#### Recruitment, inclusion and exclusion criteria

This study was carried out in accordance with the recommendations for Human Subjects Policies and Guidance of the National Institutes of Health. The protocol was approved by the Rockefeller University Hospital Institutional Review Board (IRB). All subjects gave written informed consent in accordance with the Declaration of Helsinki.

Male and female volunteers (≥18 years of age) were recruited through IRB-approved posted notices and newspaper advertisements in the community. Volunteers were required to be competent to understand study procedures and understand and sign the IRB-approved informed consent in English. The presence of uncontrolled schizophrenia or other psychotic signs during the interview were exclusion criteria. In order to focus more directly on the impact of cannabis or alcohol exposure on the odds of OD diagnoses, we excluded from this study volunteers who had a cocaine dependence diagnosis. Volunteers who had used cocaine, but did not meet the DSM-IV diagnostic criteria for cocaine dependence diagnosis were not excluded.

Persons were excluded from the normal volunteer category if they had any lifetime drug abuse or dependence diagnosis by DSM-IV criteria, or any of the following: (a) any instance of drinking to a level of intoxication during the previous 30 days, (b) any use of illicit drugs including opiates, cocaine, and amphetamines during the 30 days prior to ascertainment, (c) if they had used cannabis on more than 12 days during the 30 days prior to ascertainment, (d) had used illicit drugs (with the exception of cannabis) for at least three times a week for a period of at least 1 month, in their lifetime (40). This therefore allowed for examination of a range of normative self-exposure to cannabis and alcohol, also in the normal volunteers.

The three diagnostic groups in this study are volunteers with opioid dependence (OD), volunteers with drug diagnoses except OD, and normal volunteers. These groups are described in further detail in Table 1. In further analyses, the latter two groups were combined into an overall “not OD” group, for analyses of patterns of self-exposure to specific drugs.

**Table 1:** Description of diagnostic groups (DSM-IV criteria).

	**Volunteers with opioid dependence (OD) diagnosis**	**Volunteers with drug diagnoses, except OD**	**Normal volunteers**
Description	Volunteers with opioid dependence diagnosis, as well as other drug diagnoses (if applicable)	Volunteers with any drug diagnoses, *except* OD	Volunteers without any drug diagnoses
	Cocaine dependence diagnosis was an overall exclusion criterion for this study		

### Instruments administered during clinical interviews

All ascertainments were completed during a standardized private face-to-face interview with a licensed trained clinician (e.g., M.D., D.O., Ph.D. Psychologist, Nurse Practitioner or Registered Nurse).Volunteers underwent the SCID I/P structured interview (Version 2.0; DSM-IV criteria) (43), and received the KMSK questionnaires for maximum self-exposure to cannabis, alcohol, heroin, and cocaine (38) (see below).

### KMSK scales for maximal self-exposure to specific drugs (“KMSK score”)

The KMSK scales for cannabis, alcohol, heroin, and cocaine provide ordinal measures of maximal self-exposure, thus focusing on the period in the volunteer's life when use was the heaviest. For each drug, the scales start at a minimum “0” score, which denotes that the volunteer has not had any lifetime exposure to the drug (i.e., no use). The scores then increase in integers up to a maximum (13 for heroin and alcohol, 14 for cannabis, and 16 for cocaine) (see Table 2). The KMSK score for each drug is the composite sum of responses on three items: (a) frequency of maximal use (e.g., in times per day or per week), (b) duration of pattern of maximal use (e.g., in months or years) and (c) amount used in one day or sitting (e.g., number of alcoholic drinks or cannabis joints) (38). A separate KMSK scale is also used to characterize illicit use of prescription opioids, but was not analyzed in this study (heroin was the predominant MOP-r agonist used in this cohort). Concurrent validity of KMSK scores with the respective DSM-IV dependence diagnoses has been examined, and yielded optimal “cutpoint” scores for sensitivity and specificity (38, 39).

**Table 2:** KMSK scales for maximal self-exposure to specific drugs^a, b^.

**Drugs**	**Sub-scores**			**KMSK score range: (sum of sub-scores)**	**Optimal cutpoints (males and females combined)^h^**
	**Frequency of use^e^**	**Duration of pattern^f^**	**Amount used in a sitting or a day^g^**		
	*Range:* *never used 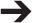 multiple daily use*	*Range:* *<6 months> 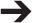 1year*	*Range:* *(see below)*		
Cannabis^c^	0 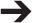 6	0 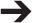 3	0 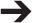 5 *None 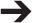 >5 joints*	0–14	10
Alcohol^c^	0 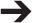 5	0 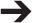 3	0 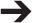 5 *None 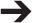 >10 drinks*	0–13	10
Heroin^d^	0 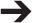 4	0 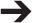 3	0 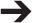 6 *None 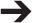 >10 doses/bags*	0–13	6
Cocaine^d^	0 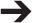 7	0 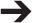 3	0 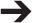 6 *$0 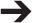 >$100* *(also converted from grams, rocks or vials)*	0–16	9

a *Ordinal integer scales; quantifying drug self-exposure at the time in the volunteer's life when use is heaviest*.

b *Scales for alcohol, heroin, and cocaine were published initially(“KMSK-1”) (38). Scales for cannabis were developed and used subsequently (“KMSK-2”) (39)*.

c *If the Frequency sub-score ≤ 2, the Duration sub-score is assigned a “0” value*.

d *If the Frequency sub-score ≤ 1, the Duration sub-score is assigned a “0” value*.

e Questionnaire text reads: “At the time in your life when you were using the most [drug], were you using it.”

f Questionnaire text reads: “How long did this pattern of [drug] use last?”

g Questionnaire text reads: “During this time when you were using the most, how much [drug] at a sitting [or day] would you typically use?”

h *Optimal cutpoints for concurrent validity in males and females combined, for the respective DSM-IV dependence diagnosis (unpublished data)*.

The KMSK scales have been used to characterize drug exposure in patients with medical and psychiatric conditions (44–46). The scales can be rapidly administered within a clinical interview (e.g., ≤5 min per drug). Each KMSK form also records age of first use, and age of onset of heaviest use (in whole years; the latter was studied herein). The four KMSK forms used in this study (i.e., for cannabis, alcohol, cocaine, and heroin), are provided in the Supplementary Materials. The full text of the scales for these and other drugs can be freely accessed: http://lab.rockefeller.edu/kreek/assets/file/KMSKquestionnaire.pdf.

### Statistical analyses

#### Missing data

If there were missing data for specific comparisons for a volunteer, the data for that volunteer was removed from analysis. The cannabis KMSK scale and the age-related items were implemented while cohort ascertainment was in progress. Therefore these items, especially for cannabis, were not available for the complete cohort.

#### Univariate analyses

Univariate analyses were carried out with GraphPad Prism software. Demographic variables (age at ascertainment, gender, and ethnicity) and KMSK scores were analyzed non-parametrically (Mann–Whitney *U*-tests or χ^2^ analyses, Kruskal–Wallis or Friedman's ANOVAs, and Dunn's *post-hoc* tests).

#### Propensity score matching

As shown in Table 3, the overall cohort had a total of *n* = 574 volunteers, of whom *n* = 94 had OD diagnoses. Of the volunteers with OD diagnoses, *n* = 89 had all KMSK scores available, and were used in the propensity score matching procedure. Propensity score matching (47), as implemented in the “MatchIt” package in R software, was applied using a 1:1 “nearest neighbor” algorithm, to minimize heterogeneity in the above demographic variables between volunteers with OD, vs. all the other volunteers. Therefore, the latter comparison group contained the volunteers with drug diagnoses except OD, and also normal volunteers (see Table 3B).

Table 3Demographics (volunteers sequentially ascertained 4/4/02-8/1/13)^a^.

**Table:** 

**(A)** Comparison of volunteers with opioid dependence (OD) diagnoses, volunteers with drug diagnoses except OD, and normal volunteers.									
**Demographics**	**Total** ***n*** = **574**	**Volunteers with OD diagnosis**^b^		**Volunteers with drug diagnoses, except OD**^C^		**Normal volunteers**^C^ **(NV)**		**Kruskal-Wallis statistic or** χ^2^ **[df];** ***p*** **value**	
		***n*** = **94**		***n*** = **187**		***n*** = **293**		
Mean age at ascertainment(SEM)		41.17	(1.23)	39.7	(0.86)	33.4	(0.70)	59.34	<0.0001
Gender	Male	65	69.1%	124	66.3%	132	45.1%	28.91 [2]	<0.0001
	Female	29	30.1%	63	33.7%	161	55.0%		
Ethnicity	African-American	25	26.6%	78	41.7%	127	43.3%	22.12 [6]	0.0012
	Caucasian	31	33.0%	59	31.6%	81	27.7%		
	Hispanic	32	34.0%	31	16.6%	47	16.0%		
	Other	6	6.4%	19	10.2%	38	13.0%

**Table:** 

**(B)** Comparison of volunteers with OD diagnosis vs. all volunteers without OD (i.e., combining volunteers with drug diagnoses except OD, and normal volunteers).						
**Demographics**	**Total** ***n*** = **574**	**Volunteers with OD diagnosis**^b^		**All volunteers without OD diagnosis**		**U or χ^2^ [df];** ***p*** **value**	
		***n*** = **94**		***n*** = **480**		
Mean age at ascertainment (SEM)		41.17	(1.23)	35.81	(0.5)	16,566	<0.0001
Gender	Male	65	69.1%	256	53.3%	7.98 [1]	0.0047
	Female	29	30.1%	224	46.7%		
Ethnicity	African-American	25	26.6%	205	42.7%	20.62 [3]	0.0001
	Caucasian	31	33.0%	140	29.2%		
	Hispanic	32	34.0%	78	16.3%		
	Other	6	6.4%	57	11.9%		

a *Cocaine dependence diagnosis was an exclusion criterion for this study. See Table 1 for further description of diagnostic groups*.

b *The same data from volunteers with OD diagnosis are presented in **(A,B)***.

c *This group combines the two right-most columns in **(A)**. The two groups presented in **(B)** are used as the input data for the propensity score matching procedure (see Table 5)*.

#### Multiple logistic regression after propensity score matching, examining cannabis, alcohol and cocaine KMSK scores as dimensional predictors of opioid dependence diagnosis

A multiple logistic regression was performed with Statistica (TIBCO) software. The predicted outcome was the presence of opioid dependence diagnosis (binary). There were *n* = 89 volunteers with and *n* = 89 without OD diagnoses in this sample, after the propensity score matching procedure, described above.

#### Alpha level for rejection of null hypotheses

For all analyses, the alpha level was *p* ≤ 0.05.

## Results

### Sample demographics

Sample Demographics are in Table 3. In order to provide a complete description of the cohort, Table 3A presents data for volunteers with OD diagnoses, volunteers with drug diagnoses except OD, and normal volunteers. Table 3B presents the same data, but the latter two groups are combined, as this is the design used in the propensity score matching procedure. See also **Table 5**, for demographic data in the two groups after execution of the propensity score matching procedure.

### Age at ascertainment

Mean age at ascertainment was greater for volunteers with OD diagnoses, and also for volunteers with drug diagnoses except OD, vs. normal volunteers (Table 3A).

### Gender

A χ^2^ analysis of gender was significant, with a greater proportion of males among volunteers with OD diagnoses, or drug diagnoses except OD, vs. normal volunteers (Table 3A).

### Ethnicity

A χ^2^ analysis of ethnicity was also significant, with a relatively greater proportion of African Americans in the normal volunteer group, and a relatively greater proportion of Caucasians and Hispanics in the OD group (Table 3A).

### Missing data

Of the 94 volunteers with OD diagnosis (see Table 3A), five were removed from further analysis, due to missing data (remaining *n* = 89). Therefore, the propensity score matching procedure used these *n* = 89 volunteers with OD diagnoses as the reference group (see below and **Table 5**). Of the 187 volunteers with a drug diagnosis except OD, 13 were removed due to missing data (remaining *n* = 174). Also, of the 293 normal volunteers, 12 were similarly removed due to missing data (remaining *n* = 281).

### Ages of onset of heaviest use of different drugs, in volunteers with opioid dependence diagnosis

The mean age of onset of heaviest use of cannabis, alcohol, cocaine and heroin are presented in Table 4, for volunteers with opioid dependence diagnosis, for whom all these data were available. A Friedman's ANOVA examining these data was significant [*F*_(4)_ = 29.22; *p* < 0.0001]. Dunn's *post-hoc* tests show that the age of onset of heaviest use of cannabis use was earlier than that for heroin or cocaine. Likewise, age of onset of heaviest use of alcohol was earlier than that for heroin or cocaine. Ages of onset of heaviest use did not differ between cannabis and alcohol, or between heroin and cocaine.

**Table 4:** Ages of onset of heaviest use of specific drugs, in volunteers with opioid dependence diagnosis (data available for each of the drugs from *n* = 47).

**Drug**	**Age of onset of heaviest use mean [95%CI]^a^**
Cannabis	18.9 [16.6–21.1]^b^
Alcohol	20.1 [18.1–22.3]^c^
Heroin	24.7 [21.9–27.5]
Cocaine	25.3 [22.6–27.9]

a *Friedman's ANOVA F_(4)_ = 29.22; p < 0.0001*.

b *Dunn's post-hoc tests: cannabis < heroin; cannabis < cocaine*.

c *Dunn's post-hoc tests: alcohol < heroin; alcohol < cocaine*.

### Propensity score matching procedure for demographic variables

As shown in Table 3B, there were demographic differences between the group with OD diagnoses and the group without OD diagnoses (the latter group being the combination of volunteers with drug diagnoses except OD, and normal volunteers). The goal of the propensity score matching procedure was to minimize the impact of the demographic differences. As is common in propensity score matching procedures, we initially utilized a multiple logistic regression to examine the demographic variables (age at ascertainment, gender, and ethnicity) as predictors of the OD diagnosis outcome. Propensity scores were then generated for each volunteer in the whole cohort, as the predicted values from this regression. These propensity scores were then entered in a matching algorithm as described in the section Materials and Methods. This algorithm selected *n* = 89 volunteers without OD diagnoses, to match the reference group of *n* = 89 volunteers with OD diagnoses. This matching procedure was effective, as confirmed by the lack of significant differences in gender, ethnicity and age at ascertainment, for the two groups (Table 5).

**Table 5:** Demographics after the propensity score matching procedure (see Table 3B for data prior to the matching procedure).

**Demographics**	**Total *n* = 178**	**Volunteers with OD diagnosis**		**Volunteers without OD diagnosis**		***U*** **or** χ^**2**^ **[df];** ***p*****-value**	
		***n*** = **89****^a^**		***n*** = **89**		
Mean age at ascertainment (SEM)		41.37	(1.30)	41.60	(1.25)	3,894	N.S. *p* = 0.85
Gender	Male	61	68.5%	56	62.9%	0.985 [1]	N.S. *p* = 0.80
	Female	28	31.5%	33	37.1%		
Ethnicity	African-American	25	28.1%	20	22.5%	0.624 [3]	N.S. *p* = 0.43
	Caucasian	27	30.3%	31	34.8%		
	Hispanic	31	34.8%	33	37.1%		
	Other	6	6.7%	5	5.6%		

a *As mentioned in text, data from 5 of the n = 94 volunteers with OD diagnoses (as described in Table 3) had to be excluded from the matching procedure, due to missing data (thus having a remaining group of n = 89 volunteers with OD diagnoses)*.

### Maximal self-exposure to cannabis, alcohol, and cocaine compared in volunteers with and without opioid dependence diagnosis, after propensity score matching

**Figure 1:**
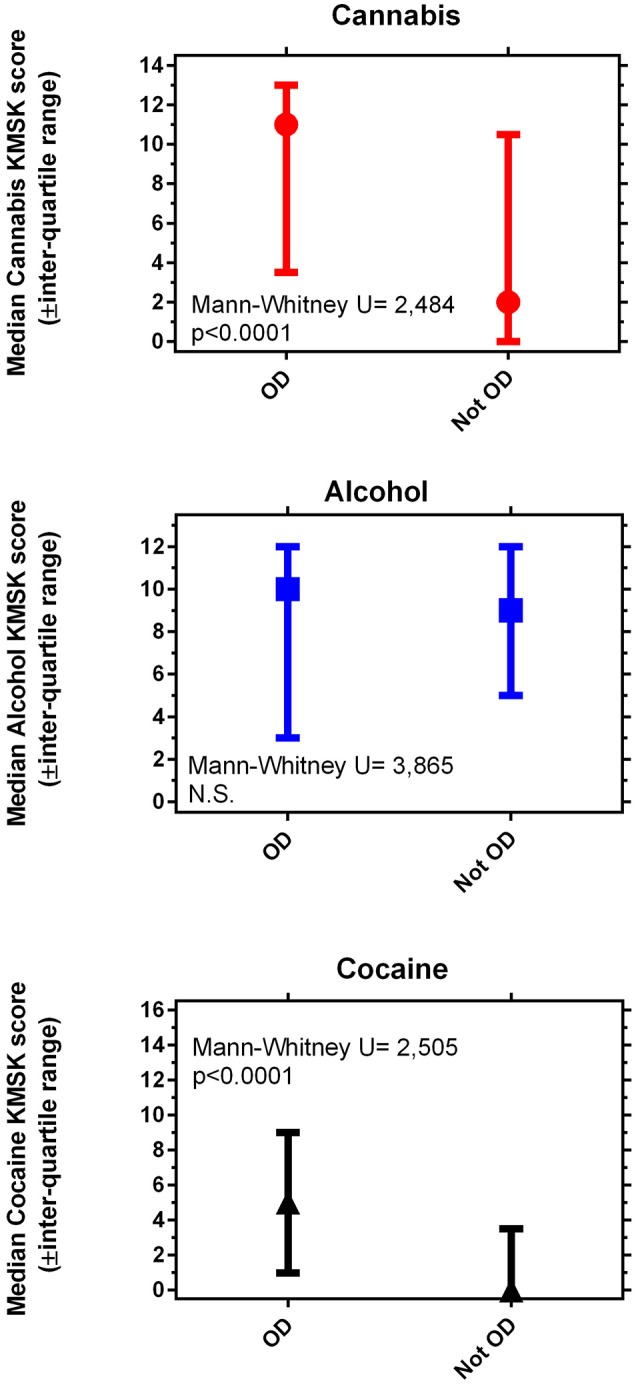
Maximal self-exposure (KMSK scores) to non-medical cannabis **(A)**, alcohol **(B)**, and cocaine **(C)**, upper, middle and lower panels, respectively, in volunteers with and without opioid dependence diagnoses (“OD” and “not OD,” respectively). The “not OD” group is the combination of volunteers with drug diagnoses except OD, and normal volunteers. The data represent two groups of *n* = 89 each, after the propensity score matching procedure (see Table 5).

Volunteers with OD had significantly greater KMSK scores for cannabis and cocaine, compared to propensity score—matched volunteers without OD (Figure 1). Alcohol KMSK scores did not differ significantly between these two groups. As expected, volunteers with an OD diagnosis had significantly greater heroin KMSK scores, compared to volunteers without this diagnosis (not shown) (38).

### Multiple logistic regression examining cannabis, alcohol, and cocaine KMSK scores as predictors of opioid dependence diagnosis, after propensity score matching

**Figure 2:**
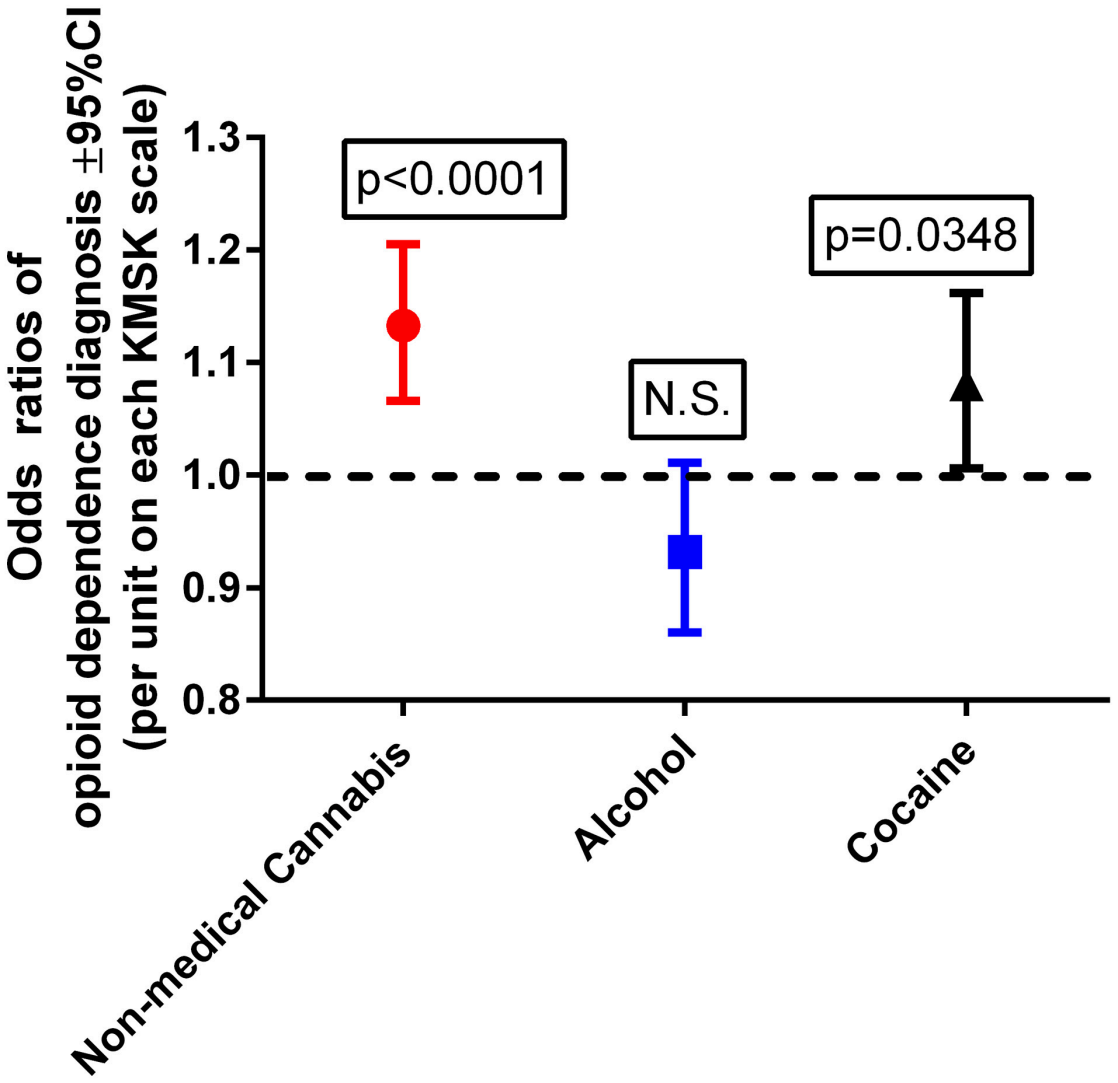
Odds ratios of opioid dependence diagnosis, with cannabis, alcohol, and cocaine KMSK scores as dimensional predictors. The ordinate is the odds ratio (±95%CL), calculated for a 1-unit score increment in each of the KMSK scales (see Table 2). The multiple logistic regression was calculated after the propensity score matching procedure (two groups, *n* = 89 each; see Table 5).

This multiple logistic regression was carried out after propensity score matching for the demographic variables, as indicated above. A Wald test for a global null hypothesis was significant [$\chi_{\left(\text{df}=3\right)}^{2}$ = 25.05; *p* < 0.0001], showing that the coefficients for the predictor variables were significantly different from 0. A Hosmer–Lemeshow test was non-significant, suggesting no evidence of lack of fit. Cannabis and cocaine KMSK scores were each detected as significant positive predictors of odds of OD diagnosis (Figure 2). By contrast, alcohol KMSK scores were not a significant predictor. Odds ratios are presented per point in each KMSK scale (score ranges in the scales are described in Table 2).

## Discussion

The impact of non-medical cannabis and alcohol use on vulnerability to develop an opioid use disorder, and to recover therefrom, has received considerable recent attention (3, 14, 15, 48). This is an area of current public health importance, given evolving trends in cannabis status across jurisdictions, the ongoing epidemic of opioid use disorders (37, 49), and the increase in prevalence of alcohol use disorders (50). However, few studies have examined dimensionally how exposure to several major drugs, especially non-medical cannabis and alcohol, impacts odds of opioid dependence diagnosis (6, 10, 35, 36, 51, 52).

### Ages of onset of heaviest use of each drug in volunteers with opioid dependence diagnosis

We found the ages of onset of heaviest use of both cannabis and alcohol preceded the onset of heaviest use of heroin, in volunteers with OD diagnosis. The ages of onset of heaviest use of cannabis and alcohol did not differ from each other, and occurred in the period of transition from adolescence to adulthood (27, 53). In this group of volunteers with an OD diagnosis, age of onset of heaviest use of cocaine occurred at a similar age as that for heroin. This overall pattern has some similarity to those previously reported (6, 53), but focuses more directly here on the ages of onset of maximal use, rather than on first use. In the context of this study, the aforementioned data provided a rationale for examining cannabis and alcohol KMSK scores as dimensional predictors of OD diagnosis. We also opted to include cocaine KMSK scores as a predictor in the multiple regression below, in order to control for differing levels of exposure to this drug that could occur in volunteers with OD diagnoses (even after exclusion of volunteers with DSM-IV cocaine dependence diagnoses).

### Demographic variables and rationale for propensity score matching

We detected significant differences in major demographic variables (age at ascertainment, gender, and ethnicity) between the different diagnostic groups. As mentioned above, this was a study of consecutive volunteers responding to advertisements in the community and in drug treatment programs, from a large ethnically diverse urban area. This may have therefore affected some of the demographic parameters of the sample. For example, epidemiological studies show that the prevalence of specific SUD can differ based on major demographic factors, including gender (54, 55). We therefore elected to carry out a propensity score matching procedure for age at ascertainment, gender, and ethnicity, prior to further analysis of maximal self-exposure to specific drugs. This matching procedure was effective in yielding groups with and without OD diagnoses, which did not differ significantly with respect to the aforementioned demographic variables.

### Maximal self-exposure to cannabis, alcohol, and cocaine, in volunteers with and without opioid dependence diagnoses, after propensity score matching

We found that both cannabis and cocaine KMSK scores were significantly greater in volunteers with OD diagnoses, vs. those without this diagnosis. Of note, the median cannabis KMSK score of volunteers with an OD diagnosis was relatively high, denoting heavy self-exposure to cannabis in this clinical group (based on a prior concurrent validity analysis with the DSM-IV cannabis dependence diagnosis 39 and unpublished data). Alcohol KMSK scores did not differ between volunteers with and without OD diagnosis, and a broad range of alcohol scores was observed in the two propensity score-matched groups. We note that cocaine KMSK scores were significantly greater in volunteers with OD than those without this diagnosis, even though a cocaine dependence diagnosis was an exclusion criterion for this study. As expected, the median cocaine KMSK score in the volunteers with OD was lower than the previously determined optimal “cutpoint” for the cocaine dependence diagnosis (38), due to the aforementioned exclusion criterion. However, the median cocaine KMSK score was even lower in the volunteers without OD (at least 50% of this group reported no lifetime cocaine use).

### Multiple logistic regression with cannabis, alcohol, and cocaine KMSK scores as predictors, after propensity score matching

After propensity score matching for age at ascertainment, gender, and ethnicity, cannabis and cocaine KMSK scores were each positive predictors of odds of an OD diagnosis. The odds in this regression were generated per 1-unit change on each KMSK scale. Therefore, it can be observed that any use of cannabis (i.e., cannabis KMSK score ≥ 1) is a predictor of increased odds of OD diagnosis (i.e., odds ratio = 1.13 per point in the cannabis KMSK scale). This study also shows that the odds of an OD diagnosis increase gradually with greater cannabis self-exposure scores. As mentioned above, recent epidemiological data from the NESARC study show that any use of cannabis at the “wave 1” time point (in 2001–2002) was a predictor of greater odds of opioid use disorder at the “wave 2” time point (in 2004–2005) (10). Other studies based on NESARC show that a dimensional measure of cannabis use (i.e., a defined frequency of use in the past year) at “wave 1” was a positive predictor of several SUDs (3), but opioid use disorder was not presented as a specific outcome in that study. Overall, several studies have examined primarily categorical measures of cannabis use as predictors of initiation of other drug use (56, 57). This study therefore provides the first rapid dimensional analysis which detects that self-exposure to non-medical cannabis is a positive predictor of odds of OD diagnosis, in a propensity score-matched sample.

Increasing cocaine self-exposure was also detected as a positive predictor of OD diagnosis, even though volunteers with cocaine dependence diagnoses were excluded from study. Therefore, even relatively smaller amounts of cocaine self-exposure are also associated with an increase in odds of OD diagnoses (further discussed below, in the “Limitations and Design Considerations” section). By contrast, alcohol KMSK scores were not a significant predictor of odds of an OD diagnosis. This study adds to the available literature on different aspects of alcohol use that may be related to opioid use disorders (9). Overall, it can be hypothesized that pharmacological or downstream neurobiological effects of cannabis, but not alcohol, can result in greater later vulnerability to opioid use disorders. An alternative interpretation, in the context of the “common liability” theory (27), is that there is a common pre-existing liability between cannabis and opioid exposure, and that alcohol does not share this liability to the same extent.

In preclinical studies, peri-adolescent exposure to Δ9-THC produces long-lasting neurobiological changes to MOP-r and dopaminergic systems, which mediate direct and indirect effects of MOP-r agonists (20, 22, 58–60). There is also evidence that some of the behavioral and downstream neurobiological effects of Δ9-THC are partially shared with MOP-r systems (25, 61). Preclinical studies show that the amount and pattern of exposure to specific drugs of abuse are critical in the emergence of underlying neurobiological changes and of addiction-like behaviors (62–65). Overall, substantial non-medical cannabis exposure in adolescence and early adulthood may result in long-lasting disruption in these and other systems, and thus result in increased vulnerability to the later development of opioid use disorders.

### Limitations and design considerations

In this study, volunteers had to recall and report aspects of their drug exposure history. The possibility that recall bias may have affected these data cannot be excluded with this type of design (66), which is very common in studies of SUD (6, 35). Recalling the age(s) at which heaviest use of a specific drug occurred is also a demand of this scale. Studies with larger cohorts, different sampling methods, as well as longitudinal studies, could be used to further extend these findings. These volunteers were ascertained prior to the passage of the relevant medical marijuana statutes for this community (31). Therefore, these findings are not necessarily relevant to the impact of medically sanctioned cannabis. Studies with later birth cohorts could also investigate possible changes to the age trajectory of exposure to different drugs, due to environmental factors (1, 67–69).

We opted here to focus on the opioid dependence diagnosis as an outcome, and to exclude volunteers who had a cocaine dependence diagnosis. This allowed us to examine more directly the impact of cannabis and alcohol self-exposure on opioid dependence diagnosis as a clinical outcome. It is known that persons with dual severe opioid and cocaine use disorder diagnoses can have a different clinical course from those with only the former diagnosis (5, 70, 71). We observed that the volunteers with OD diagnoses still had significantly greater cocaine KMSK scores than the volunteers without this diagnosis. This is not surprising, as cocaine use is relatively common in persons who use heroin (6), and can occur even in the absence of a diagnosed cocaine dependence diagnosis. As mentioned in the Methods, volunteers with cocaine *abuse* diagnoses were not excluded from study. Therefore, we included cocaine KMSK scores in the multiple logistic regression, primarily to control for the level of cocaine self-exposure.

We elected to examine two propensity score matched groups: (a) volunteers with OD, and a comparison group: (b) volunteers without OD. The latter group thus included volunteers with drug diagnoses except OD, and normal volunteers. This allowed us to have a propensity score-matched comparison group with a broad range of KMSK scores for the drugs of interest, of value for a more robust dimensional analysis (11, 38, 72).

Propensity score matching studies have become relatively frequent, and have potential strengths and limitations (3, 73). For further examination of the conclusions, we also carried out an overall multiple logistic regression with OD diagnosis as the outcome, controlling for age at ascertainment, gender and ethnicity, but without propensity score matching (i.e., including data from all volunteers in the cohort). In this overall regression, the same KMSK scores were detected as positive predictors, as in the regression in the propensity score matched groups (not shown). Therefore, the results reported above with respect to cannabis, cocaine and alcohol KMSK scores as dimensional predictors of OD diagnosis are not likely to be an artifact of the propensity score matching procedure.

## Conclusions

We detected that increasing self-exposure to non-medical cannabis was a positive predictor of odds of an OD diagnosis. We also determined that the level of maximal alcohol self-exposure *per se* was not a predictor of the OD diagnosis outcome. This is one of the few individual-level examinations in which self-exposure to cannabis and alcohol are both examined dimensionally, as predictors of a diagnosed opioid use disorder. Some recent state-wide and epidemiological studies have reported that the legalized status of medical cannabis is associated with decreases in population-wide opioid overdoses and other measures of opioid-related morbidity (14, 15). Other studies have reported divergent findings on the influence of cannabis use on treatment outcomes in opioid-dependent volunteers, possibly due to different methods used (48, 74). Non-medical cannabis use has been associated with increased probability of aberrant opioid-taking behaviors in pain patients (75), and it has also been reported recently that some persons substitute cannabis for other substances, including prescription opioids for non-medical use (76). The use of categorical vs. dimensional measures of drug use has also been suggested as a possible reason for the apparent discrepancies in this area (48). Future studies could determine whether increasing non-medical cannabis exposure, especially in adolescence and young adulthood, can result in neuro-behavioral changes that underlie greater vulnerability to opioid use disorders.

## Author contributions

EB, AM, SB, CC, and MK were involved in data collection and organization. All authors were involved in study conceptualization. EB, CC, and JC were involved in data analysis and presentation.

### Conflict of interest statement

The authors declare that the research was conducted in the absence of any commercial or financial relationships that could be construed as a potential conflict of interest.

## Abbreviations

95%CI: 95% Confidence interval
Δ9-THC: delta9-tetrahydrocannabinol
CB1-r: Cannabinoid-1 receptor
IQR: Inter-quartile range
KMSK scale: Kreek-McHugh-Schluger-Kellogg scale for maximal self-exposure to specific drugs
MOP-r: mu-opioid receptors
N.S.: Non-significant
OD: opioid dependence diagnosis (DSM-IV criteria)
OUD: Opioid use disorder (DSM-5 criteria)
ROC curve: Receiver operating characteristic curve
SUD: Substance use disorders.
